# Cardiovascular and microvascular outcomes according to vitamin D level and genetic variants among individuals with prediabetes: a prospective study

**DOI:** 10.1186/s12967-023-04557-x

**Published:** 2023-10-16

**Authors:** Pingting Zhong, Zhuoting Zhu, Yunlong Wang, Wenyong Huang, Mingguang He, Wei Wang

**Affiliations:** 1 State Key Laboratory of Ophthalmology, Zhongshan Ophthalmic Center, Guangdong Provincial Key Laboratory of Ophthalmology and Visual Science, Guangdong Provincial Clinical Research Center for Ocular Diseases, Sun Yat-sen University, Guangzhou, China; 2 Hainan Eye Hospital and Key Laboratory of Ophthalmology, Zhongshan Ophthalmic Center, Sun Yat-sen University, Haikou, China; 3 Centre for Eye Research Australia, University of Melbourne, Melbourne, Australia; 4 Zhongshan School of Medicine, Sun Yat-sen University, Guangzhou, China; 5 Experimental Ophthalmology, The Hong Kong Polytechnic University, Hong Kong, China

**Keywords:** Serum 25(OH) D, UK biobank, Prediabetes, Complications, rs2228570

## Abstract

**Background:**

Whether serum vitamin D mediate vascular diseases in prediabetic populations remains unclear. This study aimed to determine the associations between circulating 25-hydroxyvitamin D [25(OH)D] levels and vitamin D receptor (VDR) polymorphisms with the risk of macrovascular complications, including myocardial infarction and stroke, and microvascular complications such as diabetic nephropathy and retinopathy, among adults with prediabetes.

**Methods:**

Participants with prediabetes in UK Biobank were included (N = 56,387). Multivariable dose–response and Cox proportion models were used to explore the relationship of serum 25(OH)D status and the risks of vascular complications. The interaction of VDR polymorphisms with serum 25(OH)D level on risks of vascular events was also assessed.

**Results:**

During a median follow-up of 12 years, higher levels of 25(OH)D were significantly and nonlinearly associated with a lower risk of macrovascular diseases among prediabetic individuals. The adjusted hazard ratios (95% confidential interval) of serum 25(OH)D levels of ≥ 75.0 nmol/L versus < 25 nmol/L were 0.75 (0.63–0.88) for myocardial infarction, 0.74 (0.55–1.00) for stroke, 1.02 (0.60–1.74) for diabetic nephropathy, and 1.30 (0.92–1.84) for diabetic retinopathy, respectively. The rs2228570 (*FokI*) polymorphisms significantly interacted with 25(OH)D on incident myocardial infarction (P-interaction = 0.042) and stroke (P-interaction = 0.033). The individuals with serum 25(OH)D level of 50.0–74.9 nmol/L and rs2228570 (*FokI*) homozygotes had the lowest risks of vascular complications.

**Conclusions:**

Lower serum 25(OH)D levels are significantly and nonlinearly associated with an increased risk of cardiocerebrovascular diseases in prediabetic individuals, with VDR polymorphisms of rs2228570 (*FokI*) modify such associations. Monitoring a safe 25(OH)D concentration is suggested to prevent the vascular complications for prediabetes.

**Supplementary Information:**

The online version contains supplementary material available at 10.1186/s12967-023-04557-x.

## Introduction

Prediabetes is a growing public concern leading to serious vascular consequences [1, 2]. It was estimated that prediabetes affects 470 million people worldwide by 2030, who would suffer from end-organ damages in heart, brain, kidney, and eye before the diagnosis of diabetes [3, 4]. However, traditional lifestyle and drug-based interventions did no significantly reduce the risk of vascular complications among prediabetic individuals, such as myocardial infarction and stroke [5]. Development of new strategy to prevent vascular complications are urgently needed for people with prediabetes [2].

The 25-hydroxyvitamin D [25(OH)D] ranks a potent candidate for mediating vascular diseases [6–10]. Metabolites of vitamin D are essential for whole-body calcium homeostasis, and its deficiency is also related to the increased risk of diabetes, metabolic syndrome, and cardiovascular disease (CVDs) [11]. Among individuals with diabetes, lower serum 25(OH)D levels were significantly and linearly correlated with increased cardiovascular mortality [8]. Furthermore, one-unit reduction of natural log-transformed 25(OH)D was associated with a 25% higher risk of composite diabetic microvascular complications (DMC) [6]. Though they had huger number and commonly comorbidity with vitamin D deficiency, the impact of 25(OH)D on DMC risks among prediabetes remains unclear. In addition, vitamin D receptor (VDR) is widely expressed in the most tissue, including vascular smooth muscle, endothelial cells, and cardiomyocytes, as the target for vitamin D exerting physiological effects, and therefore involved in the regulation of many systemic processes [12, 13]. In the genomic mode of vitamin D action, the VDR polymorphisms are strictly associated with diseases and changes in homeostasis processes [14]. Emerging evidence suggested that the interaction between VDR polymorphisms and 25(OH)D modified the influence of 25(OH)D levels [6, 15]. Genome-wide studies have shown that the actions of 1,25(OH)_2_ D3 involve regulation of gene activity at a range of locations many kilobases from the transcription start site [14]. In addition, studies using knockout and transgenic mice have provided new insight on the physiological role of vitamin D in classical target tissues as well as evidence of extra-skeletal effects of 1,25(OH)_2_ D3 including inhibition of cancer progression, effects on the cardiovascular system, and immunomodulatory effects in certain autoimmune diseases [14]. However, whether the modification effect of VDR polymorphisms maintained for individuals with prediabetes is unclear.

To figure out these knowledge gaps, by leveraging large-scale prospective data with a median follow-up duration of 12 years from UK Biobank (UKB), we aimed to explore the correlations of serum 25(OH)D levels and vascular complications among people with prediabetes. Additionally, we further assessed whether VDR polymorphisms modify the associations of interest.

## Methods

### Study design and population

The UKB is a prospective cohort study that recruited 0.5 million adults in England, Scotland, and Wales between 2006 and 2010 [16]. The study was performed in accordance with the principle of Helsinki. All participants signed the informed consent before entering the study. In this study, participants with prediabetes and with serum 25(OH)D levels at baseline (n = 59,304) were eligible for inclusion. According to the 2021 diagnostic criteria from the American Diabetes Association guideline, prediabetes is defined with a glycosylated hemoglobin (HbA1c) level between 5.7% and 6.4% (39–47 mmol/mol), without fulfilling the diabetes criteria [17]. Those who with CVDs (n = 1489), microvascular diseases (n = 1428) at baseline were excluded (Additional file 1: Figure S1). Finally, a total of 56,387 participants with prediabetes were included in this study.

### Measurement of serum 25(OH)D

A range of biochemistry markers was measured in the blood sample collected at baseline (2006–2010). Blood samples from the UKB population were collected at the recruitment stage, separated, and stored in liquid nitrogen at − 80°C until analysis. Serum levels of 25(OH)D were quantified using a chemiluminescent immunoassay method (Liaison XL; DiaSorin, Saluggia, Italy). Calibration and quality control were conducted by the UK Biobank. The range of measurements was 5.04% and 6.14%, with an average coefficient of variation of < 10% for internal quality control and 100% assurance for external quality control. The methodology and performance are documented in the literature [6, 8, 18, 19]. The detailed information of the measurements is provided on the UK Biobank website (https://biobank.ndph.ox.ac.uk/showcase/showcase/docs/serum_biochemistry.pdf).

### Ascertainment of endpoints

Outcomes of interest included macrovascular events (myocardial infarction, stroke), and microvascular events (nephropathy, retinopathy). Data on outcome diagnoses were confirmed through codes as ICD-9, ICD-10, and OPCS-4 based on the inpatient databases, obtained from Episode Statistics-Admitted Patient Care (England), Scottish Morbidity Records-General/Acute Inpatient and Day Case Admissions (Scotland), and Patient Episode Database for Wales from 30 April 2021 (Additional file 1: Table S1). The date at which the patient first experienced any of these events during the follow-up period was defined as the date of occurrence. For each individual, the duration of follow-up for the endpoint was calculated based on the earliest date among the endpoint event, missed visit, or date of death.

### VDR Polymorphisms

Four single nucleotide polymorphisms (SNPs), namely rs731236 (*TaqI*), rs7975232 (*ApaI*), rs1544410 (*BsmI*), and rs2228570 (*FokI*), have been identified as the common allelic variants among the loss-of-function mutation in the VDR gene. *TaqI, BsmI, ApaI,* and *FokI* are four restriction endonuclease enzymes to recognize the polymorphic sites in the VDR gene. Recent attention has focused on the possible role of VDR gene variation in the development of systemic diseases, such as breast and prostate cancer, osteoarthritis, atherosclerotic coronary artery disease, diabetes, et al. Therefore, these four specific common allelic variants were analyzed in the current study for explaining whether the association of serum 25(OH)D levels and incident vascular complications could be modified by genetic variants in VDR [6].

### Assessment of covariates

The covariates of age, sex, ethnicity, educational attainment, annual household income, smoke and alcohol consumption habits (categorized as never/former/current), and medication histories were collected from questionnaires. Height, weight, blood pressure (BP), and levels of HbA1c, serum lipids and creatinine were measured by standardized procedures. Body mass index (BMI) was calculated as weight divided by height in meters squared. Hypertension was defined as having been previously diagnosed by a physician, taking antihypertensive medication, or having an average of ≥ 140/90 mmHg for three BP measurements on separate days.

### Statistical analysis

Based on the serum 25(OH)D levels, the included population was divided into four groups: < 25 nmol/L (severe deficiency); 25–50 nmol/L (moderate deficiency); 50–75 nmol/L (insufficiency); and ≥ 75 nmol/L (sufficiency) [6, 8, 18, 19]. The demographic and clinical characteristics at baseline were compared by chi-square test or ANOVA test across groups.

Cox proportional regression models were constructed to calculate hazard ratios (HRs) and their 95% confidence intervals (95% CIs) for the association between serum 25(OH)D levels and the risk of incident endpoint events. Model 1 was adjusted for sex, age, and ethnicity; Model 2 was further adjusted for income, smoking consumption status, drinking consumption habits, BMI, systolic BP, levels of total cholesterol, high-density lipoprotein, serum creatinine, medication use of vitamin D supplements, antihypertensive drugs, lipid-regulating drugs, and statins. Serum 25(OH)D levels were processed with natural logarithmic transformation when analyzed as a continuous variable.

Restricted cubic spline analysis was used to evaluate the dose–response associations of baseline serum 25(OH)D levels and the risk of incident endpoint events after adjusting for confounding variables. Akaike’s information criterion was used to determine the number of knots (four) required to fit the best approximation model. The lowest serum 25 (OH) D level was adopted as the reference, and the Wald test was used to evaluate linearity.

We also explored the risks of incident vascular complications according to joint categories of serum 25(OH)D and VDR polymorphisms. The interaction tests of serum 25(OH)D levels and each category were performed using the likelihood ratio test comparing models with and without a cross-product term.

To test the reliability and robustness of the findings, several subgroup and sensitivity analyses were performed. Firstly, stratified analyses were performed by age (< 65 years/ ≥ 65 years), sex (female/male), smoking status (never/former or current), obesity (yes/no; defined by BMI ≥ 30 kg/m^2^ or < 30 kg/m^2^), physical activity (above/below moderate vigorous physical activity recommendation). Secondly, landmark analysis was performed by excluding individuals who had endpoint events within the first year of follow-up. Thirdly, sensitivity analysis was performed by restricting for the participants with moderate or severe vitamin D deficiency (25(OH)D < 50 nmol/L).

All statistical analyses were performed using Stata 17.0 software (StataCorp, College Station, TX) and R (version 4.1.3, R Project for Statistical Computing, Vienna, Austria), setting a statistical significance of a two-sided P-value as < 0.05.

## Results

### Baseline characteristics of the participants with prediabetes

Among 56,387 subjects, 30,596 (54.3%) were females with the mean age of 59.8 ± 6.7 years, 25,791 (45.7%) were males with the mean age of 59.2 ± 7.5 years, and the median serum 25(OH)D level of whole participants was 44.4 nmol/L (interquartile range, 30.5–59.8). There were 33,702 (59.8%) participants had moderate or severe vitamin D deficiency. Participants with higher levels of 25(OH)D were older, more likely to be male, less educated, and with smoking and alcohol consumption habits (Table 1).

**Table 1:** Baseline characteristics of included participants with prediabetes by serum 25(OH)D concentration

Characteristics	All	Serum 25(OH)D concentration, nmol/L				P
		< 25.0	25.0–49.9	50.0–74.9	≥ 75.0	
N of participants	56,387	8918	24,784	17,455	5230	−
Serum 25(OH)D, nmol/L	44.4 (30.5–59.8)	19.7 (16.1–22.4)	37.7 (31.5–43.7)	60.0 (54.7–66.1)	83.8 (78.8–91.8)	< 0.001
Age, years	59.5 ± 7.1	57.3 ± 7.7	59.2 ± 7.1	60.5 ± 6.6	61.0 ± 6.4	< 0.001
Female, %	30,596 (54.3%)	4647 (52.1%)	13,483 (54.4%)	9640 (55.2%)	2826 (54.0%)	< 0.001
White, %	50,982 (90.4%)	6747 (75.7%)	22,371 (90.3%)	16,745 (95.9%)	5119 (97.8%)	< 0.001
Educational attainment, %						< 0.001
Above college	14,775 (26.2%)	2478 (27.8%)	6739 (27.2%)	4346 (24.9%)	1212 (23.2%)	
Below college	41,612 (73.8%)	6440 (72.2%)	18,045 (72.8%)	13,109 (75.1%)	4018 (76.8%)	
Annual income, %						< 0.001
< £18,000	14,265 (25.3%)	2695 (30.2%)	6293 (25.4%)	4056 (23.2%)	1221 (23.4%)	
£18,000–30999	13,308 (23.6%)	1870 (21.0%)	5809 (23.4%)	4330 (24.8%)	1299 (24.8%)	
£31,000–51999	10,712 (19.0%)	1583 (17.8%)	4817 (19.4%)	3336 (19.1%)	976 (18.7%)	
£52,000–100000	6552 (11.6%)	956 (10.7%)	2901 (11.7%)	2093 (12.0%)	602 (11.5%)	
> £100,000	1581 (2.8%)	215 (2.4%)	684 (2.8%)	505 (2.9%)	177 (3.4%)	
Missing	9969 (17.7%)	1599 (17.9%)	4280 (17.3%)	3135 (18.0%)	955 (18.2%)	
Smoker, %						< 0.001
Never	27,494 (48.8%)	4244 (47.6%)	12,142 (49.0%)	8675 (49.7%)	2433 (46.5%)	
Former	19,985 (35.4%)	2583 (29.0%)	8692 (35.1%)	6583 (37.7%)	2127 (40.7%)	
Current	8512 (15.1%)	1995 (22.3%)	3788 (15.3%)	2085 (12.0%)	644 (12.3%)	
Missing	396 (0.7%)	96 (1.1%)	162 (0.6%)	112 (0.6%)	26 (0.5%)	
Drinker, %						< 0.001
Never	3528 (6.2%)	1072 (12.0%)	1498 (6.0%)	762 (4.4%)	196 (3.8%)	
Former	2624 (4.7%)	521 (5.8%)	1221 (4.9%)	679 (3.9%)	203 (3.9%)	
Current	50,010 (88.7%)	7248 (81.3%)	21,969 (88.7%)	15,973 (91.5%)	4820 (92.1%)	
Missing	225 (0.4%)	77 (0.9%)	96 (0.4%)	41 (0.2%)	11 (0.2%)	
Body mass index, kg/m^2^	28.9 ± 5.3	30.2 ± 6.1	29.4 ± 5.4	28.1 ± 4.7	26.9 ± 4.4	< 0.001
Systolic BP, mmHg	141.3 ± 18.6	141.6 ± 18.9	141.6 ± 18.6	141.0 ± 18.4	139.8 ± 18.8	0.070
Total cholesterol, mmol/L	5.6 ± 1.1	5.7 ± 1.0	5.7 ± 1.0	5.6 ± 1.1	5.4 ± 1.1	0.192
HDL cholesterol, mmol/L	1.4 ± 0.4	1.3 ± 0.3	1.4 ± 0.3	1.4 ± 0.4	1.4 ± 0.4	< 0.001
Hemoglobin A1c, (%)	5.84 (5.76–5.98)	5.87 (5.77–6.03)	5.85 (5.76–5.99)	5.83 (5.76–5.97)	5.83 (5.76–5.94)	< 0.001
Serum creatinine, mmol/L	73.2 ± 16.1	73.4 ± 16.6	73.0 ± 16.0	73.6 ± 15.8	74.7 ± 16.6	< 0.001
Hypertension, %	17,924 (31.8%)	2915 (32.7%)	7957 (32.1%)	5379 (30.8%)	1673 (32.0%)	0.007
Vitamin D supplement, %	1136 (2.0%)	75 (0.8%)	360 (1.5%)	504 (2.9%)	197 (3.8%)	< 0.001
Antihypertensive drugs, %	8660 (15.4%)	1442 (16.2%)	3895 (15.7%)	2547 (15.6%)	776 (14.8%)	0.001
Lipid-lowering drugs, %	15,036 (26.7%)	2278 (25.5%)	6354 (25.6%)	4713 (27.0%)	1691 (32.3%)	< 0.001
Statins use, %	6941 (12.3%)	1084 (12.2%)	2993 (12.1%)	2082 (11.9%)	782 (15.0%)	< 0.001

Data are presented as mean ± standard deviation, median (interquartile range), or number (percentage, %)

*HDL*  high-density lipoprotein, *BP*  blood pressure

### Serum 25(OH)D and risks of vascular complications

During a median follow-up of 12.0 (11.4–12.8) years, we documented 2798 (5.0%) cases with incident myocardial infarction (median follow-up of 12.0 [11.2–12.8] years), 934 (1.7%) cases with incident stroke (median follow-up of 12.1 [11.3–12.8] years), 249 (0.4%) cases with nephropathy (median follow-up of 12.1 [11.4–12.8] years), and 717 (1.3%) cases with incident retinopathy (median follow-up of 12.1 [11.3–12.8] years). Compared to individuals with serum 25(OH)D level < 25.0 nmol/L, those with serum 25(OH)D level ≥ 75.0 nmol/L had significantly lower risks of developing myocardial infarction (HR = 0.75, 95% CI 0.63–0.88) and stroke (HR = 0.74, 95% CI 0.55–1.00). After fully adjusted, per 1-unit increment of log-transformed 25(OH)D level was associated with 17% (95% CI 19–24%) and 23% (95% CI 10–34%) decreased risks of incident myocardial infarction and stroke, respectively. No significant association of serum 25(OH)D levels with nephropathy (HR = 1.02, 95% CI 0.60–1.74) or retinopathy (HR = 1.30, 95% CI 0.92–1.84) was observed (Table 2). The result remained similar if HbA1c was further adjusted. (Additional file 1: Table S2).

**Table 2:** Adjusted HRs (95% CI) for serum 25(OH)D levels with vascular outcomes among participants with prediabetes in UK Biobank

Outcome and model	Serum 25(OH)D concentrations (nmol/L)				P_trend_	Log-transformed 25(OH)D	P
	< 25.0	25.0–49.9	50.0–74.9	≥ 75.0			
Myocardial infarction							
n/N of Events	497/8918	1194/24784	831/17455	276/5230		2798/56387	
Model 1	1.0 (Ref.)	0.75 (0.67, 0.83)	0.68 (0.61, 0.76)	0.73 (0.63, 0.85)	< 0.001	0.79 (0.73, 0.85)	< 0.001
Model 2	1.0 (Ref.)	0.82 (0.73, 0.92)	0.76 (0.67, 0.87)	0.75 (0.63, 0.88)	< 0.001	0.83 (0.76, 0.91)	0.001
Stroke							
n/N of Events	163/8918	406/24784	282/17455	83/5230		934/56387	
Model 1	1.0 (Ref.)	0.77 (0.64, 0.92)	0.68 (0.56, 0.83)	0.65 (0.50, 0.85)	< 0.001	0.72 (0.63, 0.83)	< 0.001
Model 2	1.0 (Ref.)	0.87 (0.71, 1.06)	0.78 (0.63, 0.98)	0.74 (0.55, 1.00)	0.018	0.77 (0.66, 0.90)	< 0.001
Diabetic nephropathy							
n/N of Events	46/8918	105/24784	70/17455	28/5230		249/56387	
Model 1	1.0 (Ref.)	0.74 (0.52, 1.06)	0.66 (0.45, 0.97)	0.86 (0.54, 1.40)	0.285	0.83 (0.63, 1.08)	0.160
Model 2	1.0 (Ref.)	0.93 (0.62, 1.38)	0.86 (0.55, 1.34)	1.02 (0.60, 1.74)	0.837	0.95 (0.71, 1.28)	0.744
Diabetic retinopathy							
n/N of Events	100/8918	334/24784	213/17455	70/5230		717/56387	
Model 1	1.0 (Ref.)	1.09 (0.87, 1.36)	0.92 (0.72, 1.17)	0.99 (0.73, 1.35)	0.315	0.88 (0.75, 1.03)	0.116
Model 2	1.0 (Ref.)	1.22 (0.94, 1.58)	1.06 (0.80, 1.40)	1.30 (0.92, 1.84)	0.929	0.99 (0.82, 1.18)	0.888

Model 1: adjusted for sex (female/male), age (continuous), and ethnicity (Whites/non-Whites); Model 2: further adjusted for income (< £18,000/£18,000–30,999/£31,000–51,999/£52,000–100,000/ > £100,000), smoking habits (never/former/current), drinking habits (never/former/current), BMI (continuous), systolic BP (continuous), total cholesterol (continuous), high-density lipoprotein (continuous), serum creatinine (continuous), and the use of vitamin D supplement (yes/no), antihypertensive drugs (yes/no), lipid-regulating drugs (yes/no), and statins (yes/no)

### Restricted cubic spline analysis

**Fig. 1:**
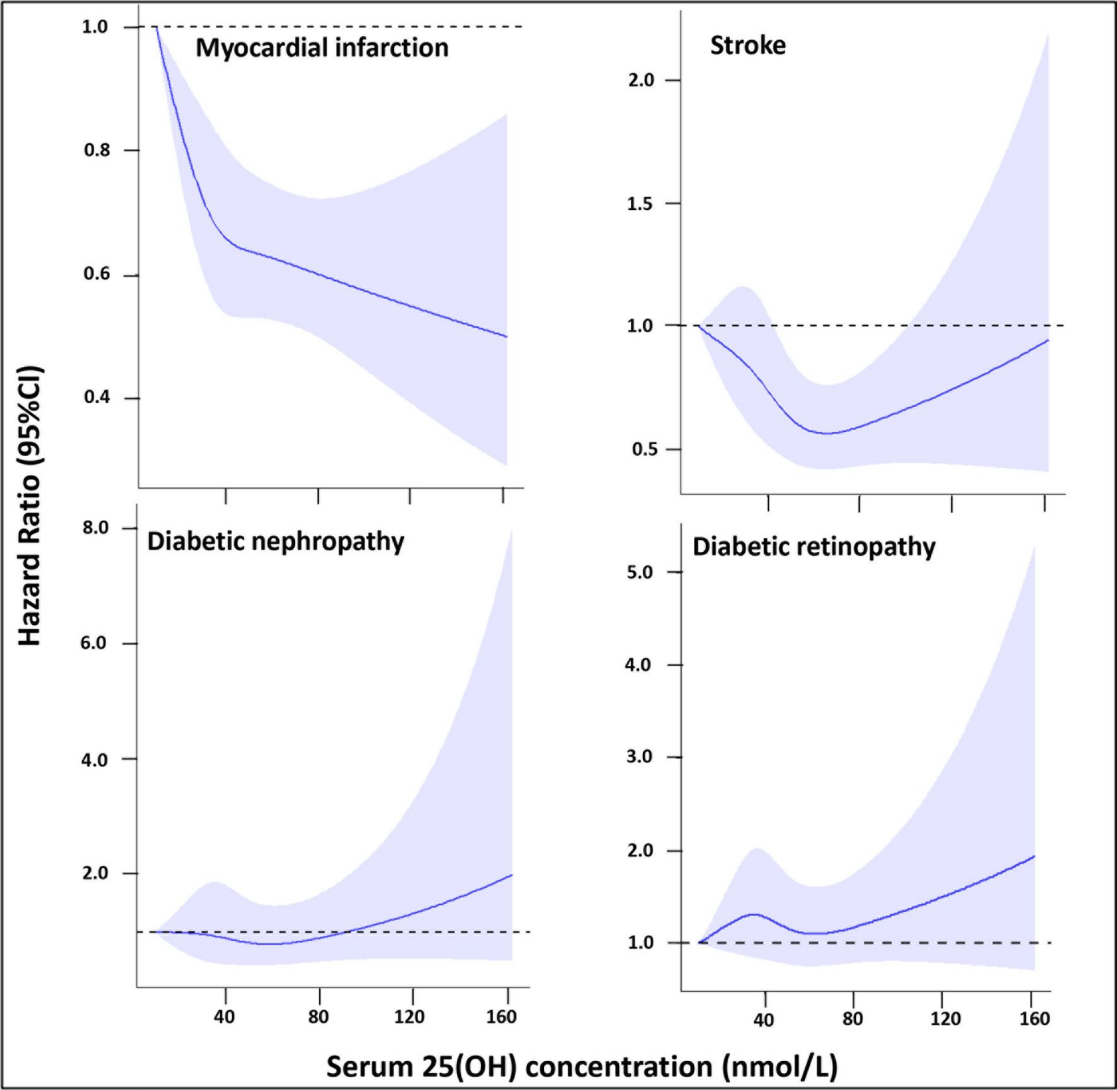
Multivariable restricted cubic spline analyses of incident events according to serum 25-hydroxyvitamin D level among in prediabetic participants. *HR* hazard ratio, *CI* confidence interval. *HRs were adjusted for sex (female/male), age (continuous), ethnicity (Whites/non-Whites), income (< £18,000/£18,000–30,999/£31,000–51,999/£52,000–100,000/ > £100,000), smoking and drinking habits (never/former/current), BMI (continuous), systolic BP (continuous), total cholesterol (continuous), high-density lipoprotein (continuous), serum creatinine (continuous), and the use of vitamin D supplement (yes/no), antihypertensive drugs (yes/no), lipid-regulating drugs (yes/no), and statins (yes/no). †P-values of non-linear were 0.013 for myocardial infarction, 0.005 for stroke, 0.281 for diabetic nephropathy, and 0.208 for diabetic retinopathy

The associations of serum 25(OH)D levels with risks of macrovascular diseases leveled off at 50 nmol/L in individuals with prediabetes (Fig. 1). Significantly non-linear dose–response associations were noted for serum 25(OH)D levels and the occurrence of myocardial infarction and stroke (both P non-linearity < 0.05) after fully adjusting for confounders. No significant dose–response relationship was observed between serum 25(OH)D levels and microvascular complications.

### Modification effects by VDR polymorphisms

For genetic analysis, major allele homozygotes (AA) of rs731236 (*TaqI*) or major allele homozygotes (CC) of rs1544410 (*BsmI*) were associated with lower concentration of 25(OH)D (Additional file 1: Table S3). However, the risks of incident vascular outcomes did not differ across their VDR genotypes (Additional file 1: Table S4).

We then tested the potential interaction between VDR polymorphisms and 25(OH)D on risks of macrovascular outcomes (Table 3). There were significant interactions between rs2228570 (*FokI*) phenotypes and 25(OH)D on the risk of incident myocardial infarction (P for interaction = 0.042) and stroke (P for interaction = 0.033). The inverse association between high 25(OH)D and myocardial infarction was more prominent among participants with rs2228570 (*FokI*) GG phenotype, whereas the reduced risk of stroke with higher 25(OH)D appeared to be more evident among participants with rs2228570 (*FokI*) AA phenotype. The result remained similar if HbA1c was further adjusted. (Additional file 1: Table S5). The people carried GG and AA allele homozygotes of rs2228570 (*FokI*) with serum 25(OH)D level of 50.0–74.9 nmol/L presented 37% (95% CI 22–48%) and 54% (95% CI 4–70%) lower risks of developing myocardial infarction and stroke, respectively, compared to those with lowest 25(OH)D level (Table 4).

**Table 3:** Interaction effects of VDR polymorphisms on the inverse association between high 25(OH)D and cardiocerebrovascular outcomes in prediabetic individuals

	Genotype	Adjusted HR (95% CI)		P _for interaction_
Myocardial infarction				
rs731236 (*TaqI*)	GG / AA	0.92 (0.74, 1.15)	0.91 (0.79, 1.06)	0.255
rs7975232 (*ApaI*)	AA / CC	0.86 (0.73, 1.01)	0.94 (0.77, 1.14)	0.776
rs1544410 (*BsmI*)	TT / CC	0.91 (0.73, 1.13)	0.90 (0.78, 1.05)	0.311
rs2228570 (*FokI*)	GG / AA	0.76 (0.66, 0.88)	0.83 (0.66, 1.03)	**0.042**
Stroke				
rs731236 (*TaqI*)	GG / AA	0.59 (0.39, 0.87)	0.92 (0.72, 1.18)	0.167
rs7975232 (*ApaI*)	AA / CC	0.57 (0.43, 0.75)	0.90 (0.66, 1.23)	0.304
rs1544410 (*BsmI*)	TT / CC	0.55 (0.38, 0.80)	0.92 (0.72, 1.18)	0.129
rs2228570 (*FokI*)	GG / AA	0.70 (0.55, 0.90)	0.66 (0.44, 0.98)	**0.033**

Bold indicates statistically significant (*P* < 0.05)

*HR*  hazard ratio, *CI*  confidence interval, *CVD*  cardiovascular disease. Cox models were adjusted for sex (female/male), age (continuous), ethnicity (Whites/non-Whites), income (< £18,000/£18,000–30,999/£31,000–51,999/£52,000–100,000/ > £100,000), smoking habits (never/former/current), drinking habits (never/former/current), BMI (continuous), systolic BP (continuous), total cholesterol (continuous), high-density lipoprotein (continuous), serum creatinine (continuous), and the use of vitamin D supplement (yes/no), antihypertensive drugs (yes/no), lipid-regulating drugs (yes/no), and statins (yes/no). HR indicated the risk for per one unit increment in serum 25(OH)D concentration

**Table 4:** The joint associations of serum 25(OH)D and genetic variants in VDR with risk of cardiovascular outcomes in prediabetes

rs2228570 (*FokI*)	25 (OH) D (nmol/L)	Myocardial infarction		Stroke	
		n/N events	HR (95%CI)	n/N Events	HR (95%CI)
GG	< 25.0	208/3706	1.0 (Ref.)	64/3706	1.0 (Ref.)
	25.0–49.9	415/9577	**0.68 (0.56, 0.81)**	153/9577	0.87 (0.63, 1.20)
	50.0–74.9	299/6619	**0.63 (0.52, 0.78)**	112/6619	0.81 (0.57, 1.16)
	≥ 75.0	102/1995	**0.68 (0.52, 0.89)**	18/1995	**0.50 (0.29, 0.86)**
AG	< 25.0	210/3969	1.0 (Ref.)	73/3969	1.0 (Ref.)
	25.0–49.9	596/11530	0.98 (0.82, 1.17)	195/11530	0.92 (0.68, 1.25)
	50.0–74.9	389/8178	0.86 (0.71, 1.04)	135/8178	0.83 (0.60, 1.14)
	≥ 75.0	132/2403	0.85 (0.66, 1.08)	51/2403	0.98 (0.66, 1.47)
AA	< 25.0	75/1173	1.0 (Ref.)	26/1173	1.0 (Ref.)
	25.0–49.9	174/3505	0.78 (0.58, 1.04)	54/3505	0.67 (0.40, 1.11)
	50.0–74.9	135/2538	0.85 (0.62, 1.16)	32/2538	**0.54 (0.30, 0.96)**
	≥ 75.0	41/795	0.66 (0.43, 1.02)	14/795	0.67 (0.32, 1.38)

Bold indicates statistically significant (*P* < 0.05)

*HR*  hazard ratio, *CI*  confidence interval, *Cox* models were adjusted for sex (female/male), age (continuous), ethnicity (Whites/non-Whites), income (< £18,000/£18,000–30,999/£31,000–51,999/£52,000–100,000/ > £100,000), smoking habits (never/former/current), drinking habits (never/former/current), BMI (continuous), systolic BP (continuous), total cholesterol (continuous), high-density lipoprotein (continuous), serum creatinine (continuous), and the use of vitamin D supplement (yes/no), antihypertensive drugs (yes/no), lipid-regulating drugs (yes/no), and statins (yes/no)

### Stratified and sensitivity analyses

Consistent and robust results were obtained from stratified analyses by age, sex, BMI, smoking and physical activity status (Additional file 1: Table S6). After excluding new-onset events in the first year of follow-up, the results remained the similar as the primary analysis (Additional file 1: Table S7). Restricting analysis for prediabetic subjects with vitamin D deficiency showed greater benefits in decreasing the risk of incident myocardial infarction (HR = 0.82, 95% CI 0.71–0.95) and stroke (HR = 0.73, 95% CI 0.57–0.93) (Additional file 1: Table S8). The results showed similar findings as the primary analysis after restricted the participants only in Whites (Additional file 1: Table S9).

## Discussion

This prospective study, which was based on 12 years of follow-up in the UKB, demonstrated that increased serum 25(OH)D levels were nonlinearly and significantly associated with lower risks of diabetic macrovascular complications (myocardial infarction, stroke) in prediabetic individuals. The VDR rs2228570 (*FokI*) polymorphisms could modify with these inverse associations. The reduced HR of macrovascular complications associated with high 25(OH)D appeared to be more prominent among participants with moderate or severe vitamin D deficiency (25(OH)D < 50 nmol/L). Series of subgroup and sensitivity analyses have confirmed the robustness of these findings.

Emerging evidence has demonstrated that serum 25(OH)D may be a potential mediator of regulating BP, developing coronary heart disease, heart failure and stroke among dysglycemic population [19, 20]. Several Mendelian randomization analyses have suggested the causal relationship between serum 25(OH)D and mortality and CVD risk [7, 21, 22]. Besides, a L-shaped non-linear relationship of genetically predicted serum 25(OH)D and CVD risks was found, with CVD risk initially decreased steeply as concentrations increasing and levelled off at 50 nmol/L approximately [22]. However, limited existing literature has included people with prediabetes and assess the effect of different serum 25(OH)D status, despite the continuum of risk across the spectrum of 25(OH)D values. Our study highlighted the beneficial effects of vitamin D concentration on macrovascular health among prediabetic population, which reached the consistent findings with previous studies on other populations.

The possible mechanisms between the inverse association of serum 25(OH)D levels and macrovascular diseases among prediabetic people may exist in several aspects. Oxidative imbalance has been correlated with the development and progression of atherosclerosis and cardiovascular outcomes. Vitamin D is known to reduce oxidative stress via the upregulation of cellular glutathione and superoxide dismutase (SOD) [11]. Therefore, the vitamin D deficiency may lead to oxidative imbalance. Specific oxidative pathways involving pro-oxidant and antioxidant enzymes may play an essential role in producing reactive oxygen species (ROS) [11]. Besides, the function of 1,25(OH)_2_D lowering BP has been demonstrated via the downregulation of renin expression and renin–angiotensin–aldosterone system (RAAS) activity, as well as the interaction with the VDR [23]. Then, vitamin D deficiency may alter pancreatic insulin secretion, peripheral insulin resistance, persistence of systemic “sterile” inflammation and immune activation, which increase the risk of the development of diabetes and its complications [24]. Besides, the active metabolite involved in fundamental processes of potential relevance to CVDs, including cell proliferation and differentiation, apoptosis, oxidative stress, membrane transport, matrix homeostasis, and cell adhesion [25]. Vitamin D also regulates BP by acting on endothelial cells and smooth muscle cells [26]. Furthermore, the endothelial diastolic dysfunction, atherosclerosis, myocardial hypertrophy, and increased endothelial inflammatory response related to low serum 25(OH)D levels [27]. In this way, the vitamin D supplement may favor the patients with chronic diseases, especially for those with vitamin D deficiency, by reducing oxidative stress and endothelial damage. Furthermore, sodium-glucose cotransporter 2 (SGLT-2) inhibitor is another important hypoglycaemic medication with anti-oxidant effects [28]. SGLT-2 inhibitors such as Empagliflozin have been shown to reduce cardiovascular mortality in patients with diabetes. One study demonstrated that Empagliflozin suppresses cardiomyocytes autosis (autophagic cell death) to confer cardioprotective effects [29]. SGLT-2 inhibitors also increase circulating levels of ketone bodies, which has been demonstrated to enhance myocardial energetics and induce reverse ventricular remodeling [30]. Since SGLT-2 could exert systemic and cardiac anti-inflammatory effects in both preclinical and clinical trials, its use for prediabetes with DOXO-induced cardiotoxicity may be a promising cardioprotective strategy [28].

Whether vitamin D supplement benefits the health outcome remained unclear, both in diabetic and general population. For instance, a randomized controlled trial (RCT) exploring the effect of vitamin D supplement on glycemic control among diabetic individuals revealed that 3-month period dosing could improve the HbA1c levels and decrease advanced oxidation protein products levels [31]. Besides, high-dose vitamin D supplement also found to improve the cardiovascular autonomic neuropathy in Type 1 diabetes [32]. However, the updated systemic review and RCTs argued the benefit of commonly used multivitamins (including vitamin D) but highlighted no effect on the all-cause mortality, cardiovascular health, and kidney function. Indeed, the benefit of vitamin supplements may vary across different dietary backgrounds, and no extra suggestion when the nutrient is sufficient [33–37]. Notably, some of these studies have typically been conducted in vitamin D-sufficient subjects [36, 38–41]. For example, in the D-Health Trial, participants had mean serum 25(OH)D levels of 115 nmol/L in the vitamin D group. The potential threshold effect for vitamin D might partly explain the non-significant findings in the previous studies [7]. Up to date, very limited evidence supports the effect of vitamin D supplement on high-risk status of prediabetes. Therefore, RCTs adopting vitamin D supplement are needed for individuals with prediabetes.

The interaction effect of genetic variants and cardiometabolic profiles may modify the relationship of vitamin D levels in prediabetic people, indicating different effect of VDR variants between diabetes and prediabetes. Among the diabetes in the UKB, both rs1544410 (*BsmI*) and rs731236 (*TaqI*) were significantly correlated with risk of microvascular complications, though they did not affect the serum 25(OH)D concentrations [6]. Furthermore, no VDR polymorphisms interacted significantly with 25(OH)D for diabetes [6]. On the contrary, this study revealed that serum 25(OH)D concentrations varied across phenotypes of rs1544410 (*BsmI*) and rs731236 (*TaqI*) among the individuals with prediabetes. Furthermore, rs2228570 (*FokI*) interacted with the 25(OH)D, with higher influence of 25(OH)D in GG and AA allele homozygotes for myocardial infarction and stroke, respectively. Among of the four SNPs, rs2228570 (*FokI*) is the only polymorphism influencing size of translated protein [42]. VDR of rs2228570 (*FokI*) has been proven associated with serious of chronic diseases, such as BP elevation and dyslipidemia, and even be served as the genetic marker for coronary artery disease [42, 43]. The functional impact of these polymorphism, TaqI, BsmI, ApaI, and FokI, have not yet been clearly described. Further studies are needed to explore the relationship of polymorphism VDR and vascular diseases [14].

Several major factors are associated with the occurrence and the progression of diabetes-related vascular diseases. For example, obesity is strongly and negatively related to serum levels of 25(OH)D [44], which may be explained by the less outdoor activity and shorter sun exposure time linked to the of the obese individuals, and thus reduce the vitamin D’s bioavailability [45]. Notably, pro-Vit D is activated to vitamin D by ultraviolet light [14]. Besides, smoking may increase RAAS activation and oxidative stress, which may contribute to endothelial dysfunction in these people [11]. Therefore, we further explored the interaction effects of these risk factors on the serum 25(OH)D levels on the vascular endpoints, with the results revealed no significant interaction effect.

In our study, the non-significant associations between 25(OH) and microvascular complications were consistent with the previous observational and interventional studies [35, 36, 40, 46]. For example, in the Vitamin D and Omega-3 Trial (VITAL), the use of vitamin D supplement did not help to prevent or delay the renal function decline among diabetic people [35]. In the Fenofibrate Intervention and Event Lowering in Diabetes (FIELD) study, the effect of serum 25(OH)D on microvascular disease was attributable to the seasonality, hs-CRP, and physical activity level [10]. This study advanced the studies of diabetes and revealed that 25(OH)D had minimal effect on kidney and eye disease among prediabetic individuals.

Our study may have its clinical implication for people with prediabetes. To date, limited evidence was available on screening for vitamin D deficiency improve health outcomes [46]. The latest USPSTF guidelines recommended screening for prediabetes in adults aged 35 to 70 years who have overweight or obesity [47, 48]. The ADA guidelines also stressed the lifestyle intervention for the management of prediabetes and diabetes [49]. However, no significant trend pertaining to the reduction of myocardial infarction existed and only borderline reduced risk on stroke was noted in the lifestyle-drug-intervention group for prediabetic individuals [5]. Our study highlighted the beneficial effects of maintain vitamin D at a safe level (e.g., > 50 nmol/L), and the incorporation of monitoring 25(OH)D into current guidelines might be helpful for management of prediabetes.

The biggest novelty of this study is the target people with prediabetes and their VDR polymorphisms interaction effect. Though the association between 25(OH)D and vascular complications, as well as their interaction with VDR polymorphisms have been extensively explored, most of them focused on the status of diabetes. While prediabetes is also a growing public concern leading to serious vascular consequences, limited study has focused the impact of 25(OH) on this specific stage and their vascular complication. Therefore, our study tried to give a comprehensive understanding of the relationship between 25(OH) and vascular complications and their interaction effect by VDR polymorphisms, based on the large-scale prospective cohort study.

This study has several strengths. Firstly, our findings were based on large-scale prospective data, with long-term follow-up duration. A special population of prediabetes were targeted, which is considered as a high-risk status of developing diabetes and various cardiometabolic dysfunction, to figure out whether serum vitamin D status could affect the risks of vascular health among prediabetic individuals. Then, genetic variants with popular VDR SNPs were further used to perform the modification effects of serum 25(OH)D concentrations on vascular outcomes. Furthermore, we additionally explored the interaction effects of cardiometabolic profiles, such as age, sex, BMI, on these results. Lastly, a series of stratified and sensitivity analyses were performed to enhance our primary findings.

We also acknowledged some limitations. For the exposure of vitamin D, the measurement of serum 25(OH)D in this study was based on an electrochemiluminescence immunoassay, but not on a gold standard method of liquid chromatography mass spectrometry [50]. Then, while the form of 25(OH)D is generally accepted as the best indicator for assessing the total body vitamin D stores [51], in fact, 1,25(OH)_2_D (calcitriol) is the activated form of vitamin D binding to VDR. However, the measurement of 1,25(OH)_2_D was unavailable among these study population. Besides, this study did not examine the dynamic changes in serum 25(OH)D levels, which limited us to dynamically assess the dynamic effect of serum 25(OH)D status on the risk of vascular complications. Hence, further studies are warranted to explore the association between low serum 25(OH)D levels and the increased likelihood of macrovascular complications, as well as to determine whether serum 25(OH)D can serve as a crucial biomarker for macrovascular complications in individuals with prediabetes.

Furthermore, although we did adjust the covariate of medication use of vitamin D supplement, it seemed that we lacked the data of specific daily intake dose. People with different baseline vitamin D levels age may be recommended with different dosing according to Endocrine Society’s clinical practice guidelines [52], and the actual daily vitamin D supplement dosing may be helpful if was further taken into consideration. For the population selection, medication use of statins may improve lipid parameters and therefore partially interfere with the correlation of vitamin D status and lipid profile. Since this study targeted the prediabetic population and their actual vascular outcomes, we then decided not to exclusively select the participants. For the endpoint events, we only presented the effects on macro- and microvascular diseases of myocardial infarction, stroke, diabetic nephropathy, and DR. Future studies may include more subtypes of vascular disease to delineate the effects of vitamin D on risks of various conditions.

## Conclusions

In the UK prediabetic population, lower serum 25(OH)D levels are significantly and nonlinearly associated with an increased risk of macrovascular complications. The VDR polymorphisms could modify with the influence of serum 25(OH)D, and rs2228570 (*FokI*) appeared with the strongest modification effect. Serum 25(OH)D levels may serve as an essential biomarker of macrovascular complications for prediabetic people and monitoring a safe 25(OH)D concentration is suggested to prevent the macrovascular diseases for prediabetes.

### Supplementary Information

**Additional file 1: **Figure S1. Flowchart of the study participants. **Table S1.** ICD and OPCS codes for vascular diseases. 3. **Table S2.** Adjusted HRs (95% CI) for serum 25(OH)D levels with vascular outcomes among participants with prediabetes in UK Biobank. **Table S3.** Serum 25(OH)D concentrations according to different VDR genotypes among patients with prediabetes in UK Biobank.5. **Table S4.** Multivariable-adjusted HRs (95% CIs) for associations of VDR polymorphisms and vascular complications in prediabetic participants in UK Biobank. **Table S5.** Interaction effects of VDR polymorphisms on the inverse association between high 25(OH)D and cardiocerebrovascular outcomes in prediabetic individuals (additional Model) 7. **Table S6.** Hazard ratios (95% CI) of serum 25(OH)D concentration and incident cardiocerebrovascular complications in prediabetic individuals in UK Biobank: stratified analysis.8. **Table S7.** Adjusted HRs (95%CI) of serum 25(OH)D level with outcomes in prediabetic participants after excluding outcomes occurred within first year of follow up.9. **Table S8.** Adjusted HRs (95%CI) of serum 25(OH)D level with outcomes in prediabetic participants limited in vitamin D deficiency population (<50.0 nmol/L).10. **Table S9.** Interaction effects of VDR polymorphisms on the inverse association between high 25(OH)D and cardiocerebrovascular outcomes in prediabetic individuals: restricted in Whites.11.

## Data Availability

Data and materials are available via UK Biobank at http://www.ukbiobank.ac.uk/.

## Abbreviations

25(OH)D: 25-Hydroxyvitamin D
BMI: Body mass index
HR: Hazard ratio
CI: Confidence interval
CVD: Cardiovascular disease
DR: Diabetic retinopathy
UKB: UK Biobank
