# Supplementary material for: Cardiovascular and microvascular outcomes according to vitamin D level and genetic variants among individuals with prediabetes: a prospective study
Source: J Transl Med. 2023 Oct 16;21:724. doi: 10.1186/s12967-023-04557-x (PMC10577927; doi:10.1186/s12967-023-04557-x)
Supplement: Supplementary file 1 — Additional file 1: Figure S1. Flowchart of the study participants. Table S1. ICD and OPCS codes for vascular diseases. 3. Table S2. Adjusted HRs (95% CI) for serum 25(OH)D levels with vascular outcomes among participants with prediabetes in UK Biobank. Table S3. Serum 25(OH)D concentrations according to different VDR genotypes among patients with prediabetes in UK Biobank.5. Table S4. Multivariable-adjusted HRs (95% CIs) for associations of VDR polymorphisms and vascular complications in prediabetic participants in UK Biobank. Table S5. Interaction effects of VDR polymorphisms on the inverse association between high 25(OH)D and cardiocerebrovascular outcomes in prediabetic individuals (additional Model) 7. Table S6. Hazard ratios (95% CI) of serum 25(OH)D concentration and incident cardiocerebrovascular complications in prediabetic individuals in UK Biobank: stratified analysis.8. Table S7. Adjusted HRs (95%CI) of serum 25(OH)D level with outcomes in prediabetic participants after excluding outcomes occurred within first year of follow up.9. Table S8. Adjusted HRs (95%CI) of serum 25(OH)D level with outcomes in prediabetic participants limited in vitamin D deficiency population (<50.0 nmol/L).10. Table S9. Interaction effects of VDR polymorphisms on the inverse association between high 25(OH)D and cardiocerebrovascular outcomes in prediabetic individuals: restricted in Whites.11. [file 12967_2023_4557_MOESM1_ESM.docx]

**Additional file materials**

**Additional file Figure: 1**

**Additional file Tables: 9**

[Additional file Figure 2](#_Toc139965857)

[Figure S1. Flowchart of the study participants. 2](#_Toc139965858)

[Additional file Table 3](#_Toc139965859)

[Table S1. ICD and OPCS codes for vascular diseases. 3](#_Toc139965860)

[Table S2. Adjusted HRs (95% CI) for serum 25(OH)D levels with vascular outcomes among participants with prediabetes in UK Biobank. 4](#_Toc139965861)

[Table S3. Serum 25(OH)D concentrations according to different VDR genotypes among patients with prediabetes in UK Biobank. 5](#_Toc139965862)

[Table S4. Multivariable-adjusted HRs (95% CIs) for associations of VDR polymorphisms and vascular complications in prediabetic participants in UK Biobank. 6](#_Toc139965863)

[Table S5. Interaction effects of VDR polymorphisms on the inverse association between high 25(OH)D and cardiocerebrovascular outcomes in prediabetic individuals (additional Model). 7](#_Toc139965864)

[Table S6. Hazard ratios (95% CI) of serum 25(OH)D concentration and incident cardiocerebrovascular complications in prediabetic individuals in UK Biobank: stratified analysis. 8](#_Toc139965865)

[Table S7. Adjusted HRs (95%CI) of serum 25(OH)D level with outcomes in prediabetic participants after excluding outcomes occurred within first year of follow up. 9](#_Toc139965866)

[Table S8. Adjusted HRs (95%CI) of serum 25(OH)D level with outcomes in prediabetic participants limited in vitamin D deficiency population (<50.0 nmol/L). 10](#_Toc139965867)

[Table S9. Interaction effects of VDR polymorphisms on the inverse association between high 25(OH)D and cardiocerebrovascular outcomes in prediabetic individuals: restricted in Whites. 11](#_Toc139965868)

# Additional file Figure

## Figure S1. Flowchart of the study participants.

# Additional file Table

## Table S1. ICD and OPCS codes for vascular diseases.

| **Endpoints** | **ICD–9** | **ICD–10** | **OPCS4** |
| --- | --- | --- | --- |
| Myocardial infarction | 410, 411, 412 | I21, I22, I23, I24.1, I25.2 |  |
| Stroke | 433–435; 430–432 | I63, I65, I66; I60–I62 |  |
| Diabetic nephropathy | 585 | E85.3, N16.5, N18.0, N18.5, Q60.1, T82.4, T86.1, Y60.2, Y61.2, Y62.2, Y84.1, Z49.0, Z49.1, Z49.2, Z94.0, Z99.2 | L74.1, L74.2, L74.3, L74.4, L74.5, L74.6, L74.8, L74.9, M01.2, M01.3, M01.4, M01.5, M01.8, M01.9, M02.3, M08.4, M17.2, M17.4, M17.8, M17.9, X40.1, X40.2, X40.3, X40.4, X40.5, X40.6, X40.7, X40.8, X40.9, X41.1, X41.2, X41.8, X41.9, X42.1, X42.8, X42.9, X43.1 |
| Diabetic retinopathy | 362.01–362.07; 379.23, 362.81,361.0, 361.8, 361.9, 362.53, 369 | H36.0, E09.319, E08.319, E13.319, E10.319, E11.319 | C89x, C82x, C79x |

## Table S2. Adjusted HRs (95% CI) for serum 25(OH)D levels with vascular outcomes among participants with prediabetes in UK Biobank.

| **Outcome and model** | **Serum 25(OH)D concentrations (nmol/L)** | | | | **P _trend_** | **Log-transformed 25(OH)D** | **P** |
| --- | --- | --- | --- | --- | --- | --- | --- |
|  | <25.0 | 25.0–49.9 | 50.0–74.9 | ≥75.0 |  |  |  |
| **Myocardial infarction** |  |  |  |  |  |  |  |
| n/N of Events | 497/8918 | 1194/24784 | 831/17455 | 276/5230 |  | 2798/56387 |  |
| Additional Model | 1.0 (Ref.) | 0.82 (0.73, 0.92） | 0.76 (0.67, 0.86) | 0.75 (0.63, 0.88) | <0.001 | 0.83 (0.76, 0.91) | <0.001 |
| **Stroke** |  |  |  |  |  |  |  |
| n/N of Events | 163/8918 | 406/24784 | 282/17455 | 83/5230 |  | 934/56387 |  |
| Additional Model | 1.0 (Ref.) | 0.87 (0.71, 1.06) | 0.77 (0.62, 0.96) | 0.74 (0.55, 1.00) | 0.015 | 0.77 (0.66, 0.90) | 0.001 |
| **Diabetic nephropathy** |  |  |  |  |  |  |  |
| n/N of Events | 46/8918 | 105/24784 | 70/17455 | 28/5230 |  | 249/56387 |  |
| Additional Model | 1.0 (Ref.) | 0.89 (0.59, 1.32) | 0.83 (0.54, 1.29) | 1.01 (0.60, 1.73) | 0.867 | 0.95 (0.70, 1.28) | 0.730 |
| **Diabetic retinopathy** |  |  |  |  |  |  |  |
| n/N of Events | 100/8918 | 334/24784 | 213/17455 | 70/5230 |  | 717/56387 |  |
| Additional Model | 1.0 (Ref.) | 1.20 (0.93, 1.56) | 1.04 (0.79, 1.38) | 1.28 (0.91, 1.81) | 0.659 | 0.989 (0.82, 1.18) | 0.860 |

Additional Model: adjusted for sex (female/male), age (continuous), and ethnicity (Whites/non-Whites), income (<£18,000/£18,000–30,999/£31,000–51,999/£52,000–100,000/>£100,000), smoking habits (never/former/current), drinking habits (never/former/current), BMI (continuous), systolic BP (continuous), total cholesterol (continuous), high-density lipoprotein (continuous), serum creatinine (continuous), and the use of vitamin D supplement (yes/no), antihypertensive drugs (yes/no), lipid-regulating drugs (yes/no), statins (yes/no), and hemoglobin A1c (continuous).

## Table S3. Serum 25(OH)D concentrations according to different VDR genotypes among patients with prediabetes in UK Biobank.

|  | **N of participants** | **Serum 25(OH)D, nmol/L** | **P _trend_** |
| --- | --- | --- | --- |
| rs731236 (*TaqI*) |  |  | 0.015 |
| AA | 20819 | 43.6 (30.0–59.2) |  |
| AG | 26356 | 44.6 (30.7–60.3) |  |
| GG | 8813 | 45.5 (31.4–60.3) |  |
| rs7975232 (*ApaI*) |  |  | 0.117 |
| CC | 12432 | 44.6 (30.8–59.8) |  |
| AC | 27551 | 44.3 (30.6–59.9) |  |
| AA | 16005 | 44.4 (30.2–59.6) |  |
| rs1544410 (*BsmI*) |  |  | 0.007 |
| CC | 20207 | 43.8 (30.3–59.3) |  |
| CT | 26608 | 44.5 (30.6–60.1) |  |
| TT | 9173 | 45.1 (30.9–60.0) |  |
| rs2228570 (*FokI*) |  |  | 0.958 |
| AA | 8011 | 45.3 (31.3–60.6) |  |
| AG | 26080 | 44.7 (30.9–59.8) |  |
| GG | 21897 | 43.8 (29.8–59.5) |  |

Data are presented as median (interquartile range).

## Table S4. Multivariable-adjusted HRs (95% CIs) for associations of VDR polymorphisms and vascular complications in prediabetic participants in UK Biobank.

|  | **Myocardial infarction** | **Stroke** | **Diabetic nephropathy** | **Diabetic retinopathy** |
| --- | --- | --- | --- | --- |
| **rs731236 (*TaqI*)** |  |  |  |  |
| GG | 1.0 (Ref.) | 1.0 (Ref.) | 1.0 (Ref.) | 1.0 (Ref.) |
| AG | 1.01 (0.90, 1.14) | 1.03 (0.84, 1.27) | 0.77 (0.53, 1.12) | 0.97 (0.77, 1.21) |
| AA | 1.02 (0.90, 1.15) | 1.17 (0.95, 1.44) | 0.83 (0.56, 1.22) | 0.92 (0.73, 1.16) |
| **rs7975232 (*ApaI*)** |  |  |  |  |
| AA | 1.0 (Ref.) | 1.0 (Ref.) | 1.0 (Ref.) | 1.0 (Ref.) |
| AC | 1.05 (0.95, 1.15) | 1.01 (0.86, 1.19) | 1.34 (0.96, 1.88) | 1.06 (0.88, 1.28) |
| CC | 0.97 (0.87, 1.09) | 1.13 (0.93, 1.37) | 1.40 (0.95, 2.07) | 0.92 (0.73, 1.15) |
| **rs1544410 (*BsmI*)** |  |  |  |  |
| TT | 1.0 (Ref.) | 1.0 (Ref.) | 1.0 (Ref.) | 1.0 (Ref.) |
| CT | 1.02 (0.91, 1.14) | 0.99 (0.81, 1.21) | 0.79 (0.54, 1.15) | 1.06 (0.84, 1.32) |
| CC | 1.01 (0.90, 1.14) | 1.14 (0.93, 1.40) | 0.86 (0.59, 1.27) | 0.97 (0.77, 1.23) |
| **rs2228570 (*FokI*)** |  |  |  |  |
| GG | 1.0 (Ref.) | 1.0 (Ref.) | 1.0 (Ref.) | 1.0 (Ref.) |
| AG | 1.07 (0.98, 1.17) | 1.10 (0.95, 1.28) | 0.84 (0.63, 1.12) | 0.92 (0.77, 1.09) |
| AA | 1.12 (0.99, 1.26) | 1.01 (0.81, 1.25) | 0.83 (0.54, 1.29) | 1.06 (0.84, 1.35) |

HR=hazard ratio; CI=confidence interval; CVD=cardiovascular disease; Ref=reference.

HRs were adjusted for sex (female/male), age (continuous), ethnicity (Whites/non-Whites), income (<£18,000/£18,000–30,999/£31,000–51,999/£52,000–100,000/>£100,000), smoking and drinking habits (never/former/current), BMI (continuous), systolic BP (continuous), total cholesterol (continuous), high-density lipoprotein (continuous), serum creatinine (continuous), vitamin D supplement (yes/no), and the use of antihypertensive drugs (yes/no), lipid-regulating drugs (yes/no), and statins (yes/no).

## Table S5. Interaction effects of VDR polymorphisms on the inverse association between high 25(OH)D and cardiocerebrovascular outcomes in prediabetic individuals (additional Model).

|  | **Genotype** | **Adjusted HR (95% CI)** | | **P _for_ _interaction_** |
| --- | --- | --- | --- | --- |
| **Myocardial infarction** |  |  |  |  |
| rs731236 (*TaqI*) | GG / AA | 0.92 (0.73, 1.15) | 0.92 (0.79, 1.06) | 0.357 |
| rs7975232 (*ApaI*) | AA / CC | 0.86 (0.73, 1.01) | 0.94 (0.77, 1.14) | 0.735 |
| rs1544410 (*BsmI*) | TT / CC | 0.90 (0.73, 1.13) | 0.90 (0.78, 1.05) | 0.327 |
| rs2228570 (*FokI*) | GG / AA | 0.77 (0.66, 0.89) | 0.83 (0.66, 1.03) | **0.050** |
| **Stroke** |  |  |  |  |
| rs731236 (*TaqI*) | GG / AA | 0.59 (0.40, 0.87) | 0.93 (0.72, 1.18) | 0.150 |
| rs7975232 (*ApaI*) | AA / CC | 0.57 (0.43, 0.76) | 0.92 (0.67, 1.26) | 0.115 |
| rs1544410 (*BsmI*) | TT / CC | 0.55 (0.38, 0.81) | 0.93 (0.72, 1.19) | **0.018** |
| rs2228570 (*FokI*) | GG / AA | 0.72 (0.56, 0.93) | 0.66 (0.45, 0.99) | **0.002** |

HR=hazard ratio; CI=confidence interval; CVD=cardiovascular disease. Cox models were adjusted for sex (female/male), age (continuous), ethnicity (Whites/non-Whites), income (<£18,000/£18,000–30,999/£31,000–51,999/£52,000–100,000/>£100,000), smoking habits (never/former/current), drinking habits (never/former/current), BMI (continuous), systolic BP (continuous), total cholesterol (continuous), high-density lipoprotein (continuous), serum creatinine (continuous), and the use of vitamin D supplement (yes/no), antihypertensive drugs (yes/no), lipid-regulating drugs (yes/no), statins (yes/no), and hemoglobin A1c (continuous). HR indicated the risk for per one unit increment in serum 25(OH)D concentration.

## Table S6. Hazard ratios (95% CI) of serum 25(OH)D concentration and incident cardiocerebrovascular complications in prediabetic individuals in UK Biobank: stratified analysis.

|  | **Myocardial infarction** | | **Stroke** | |
| --- | --- | --- | --- | --- |
|  | **HR (95%CI)** | **P _interaction_** | **HR (95%CI)** | **P _interaction_** |
| **Age** |  | 0.485 |  | 0.156 |
| <65 years | 0.81 (0.72, 0.91) |  | 0.68 (0.55, 0.85) |  |
| ≥65 years | 0.88 (0.76, 1.01) |  | 0.90 (0.72, 1.12) |  |
| **Sex** |  | 0.778 |  | 0.294 |
| Female | 0.85 (0.72, 1.00) |  | 0.78 (0.63, 0.97) |  |
| Male | 0.82 (0.74, 0.91) |  | 0.76 (0.61, 0.93) |  |
| **Body mass index** |  | 0.823 |  | 0.554 |
| <30 kg/m^2^ | 0.81 (0.72, 0.91) |  | 0.74 (0.61, 0.90) |  |
| ≥30 kg/m^2^ | 0.86 (0.75, 0.98) |  | 0.82 (0.65, 1.05) |  |
| **Smoking status** |  | 0.396 |  | 0.240 |
| Never | 0.98 (0.83, 1.15) |  | 0.76 (0.59, 0.98) |  |
| Current/former | 0.76 (0.69, 0.85) |  | 0.72 (0.60, 0.88) |  |
| **MVPA** |  | 0.214 |  | 0.573 |
| Below | 0.85 (0.74, 0.98) |  | 0.86 (0.67, 1.10) |  |
| Above | 0.82 (0.71, 0.96) |  | 0.68 (0.53, 0.89) |  |

MVPA=moderate/vigorous physical activity recommendation. HRs were adjusted for sex (female/male), age (continuous), ethnicity (Whites/non-Whites), income (<£18,000/£18,000–30,999/£31,000–51,999/£52,000–100,000/>£100,000), smoking and drinking habits (never/former/current), BMI (continuous), systolic BP (continuous), total cholesterol (continuous), high-density lipoprotein (continuous), serum creatinine (continuous), and the use of vitamin D supplement (yes/no), antihypertensive drugs (yes/no), lipid-regulating drugs (yes/no), and statins (yes/no). HR indicated the risk for per one unit increment in serum 25(OH)D concentration.

## Table S7. Adjusted HRs (95%CI) of serum 25(OH)D level with outcomes in prediabetic participants after excluding outcomes occurred within first year of follow up.

|  | **Serum 25(OH)D concentrations (nmol/L)** | | | | **P _trend_** | **Log-transformed 25(OH)D** | **P** |
| --- | --- | --- | --- | --- | --- | --- | --- |
|  | **<25.0** | **25.0–49.9** | **50.0–74.9** | **≥75.0** |  |  |  |
| **Myocardial infarction** |  |  |  |  |  |  |  |
| Model 1 | 1.0 (Ref.) | 0.75 (0.68, 0.84) | 0.67 (0.60, 0.75) | 0.73 (0.63, 0.85) | <0.001 | 0.78 (0.72, 0.85) | <0.001 |
| Model 2 | 1.0 (Ref.) | 0.83 (0.73, 0.93) | 0.76 (0.67, 0.87) | 0.75 (0.64, 0.89) | <0.001 | 0.83 (0.76, 0.91) | <0.001 |
| **Stroke** |  |  |  |  |  |  |  |
| Model 1 | 1.0 (Ref.) | 0.77 (0.64, 0.93) | 0.71 (0.58, 0.87) | 0.66 (0.50, 0.87) | 0.001 | 0.74 (0.64, 0.85) | <0.001 |
| Model2 | 1.0 (Ref.) | 0.87 (0.71, 1.08) | 0.80 (0.63, 1.00) | 0.72 (0.53, 0.97) | 0.017 | 0.76 (0.65, 0.89) | <0.001 |
| **Diabetic nephropathy** |  |  |  |  |  |  |  |
| Model 1 | 1.0 (Ref.) | 0.75 (0.52, 1.08) | 0.70 (0.48, 1.04) | 0.93 (0.57, 1.52) | 0.533 | 0.89 (0.68, 1.17) | 0.410 |
| Model 2 | 1.0 (Ref.) | 0.92 (0.61, 1.38) | 0.91 (0.58, 1.42) | 1.07 (0.62, 1.83) | 0.889 | 1.02 (0.76, 1.38) | 0.898 |
| **Diabetic retinopathy** |  |  |  |  |  |  |  |
| Model 1 | 1.0 (Ref.) | 1.04 (0.82, 1.32) | 0.90 (0.70, 1.15) | 0.89 (0.64, 1.24) | 0.170 | 0.85 (0.72, 1.00) | 0.054 |
| Model 2 | 1.0 (Ref.) | 1.15 (0.88, 1.50) | 1.02 (0.76, 1.36) | 1.16 (0.81, 1.67) | 0.928 | 0.95 (0.79, 1.15) | 0.604 |

CI=confidence interval; Ref=reference. Participants with new-onset myocardial infarction (n=129), stroke (n=63), diabetic nephropathy (n=31), and diabetic retinopathy (n=197) in the first year of follow-up were excluded. Model 1: adjusted for sex (female/male), age (continuous), and ethnicity (Whites/non-Whites). Model 2: further adjusted for income (<£18,000/£18,000–30,999/£31,000–51,999/£52,000–100,000/>£100,000), smoking and drinking habits (never/former/current), BMI (continuous), systolic BP (continuous), total cholesterol (continuous), high-density lipoprotein (continuous), serum creatinine (continuous), vitamin D supplement (yes/no), and the use of antihypertensive drugs (yes/no), lipid-regulating drugs (yes/no), and statins (yes/no).

## Table S8. Adjusted HRs (95%CI) of serum 25(OH)D level with outcomes in prediabetic participants limited in vitamin D deficiency population (<50.0 nmol/L).

|  | **Log-transformed 25(OH)D** | **P** |
| --- | --- | --- |
| **Myocardial infarction** |  |  |
| n/N of Events | 1691/33702 |  |
| Model 1 | 0.70 (0.61, 0.80) | <0.001 |
| Model 2 | 0.82 (0.71, 0.95) | 0.008 |
| **Stroke** |  |  |
| n/N of Events | 569/33702 |  |
| Model 1 | 0.65 (0.52, 0.82) | <0.001 |
| Model2 | 0.73 (0.57, 0.93) | 0.011 |
| **Diabetic nephropathy** |  |  |
| n/N of Events | 151/33702 |  |
| Model 1 | 0.70 (0.45, 1.09) | 0.111 |
| Model 2 | 0.82 (0.50, 1.35) | 0.435 |
| **Diabetic retinopathy** |  |  |
| n/N of Events | 434/33702 |  |
| Model 1 | 0.96 (0.73, 1.26) | 0.759 |
| Model 2 | 1.07 (0.79, 1.46) | 0.651 |

Model 1: adjusted for sex (female/male), age (continuous), and ethnicity (Whites/non-Whites). Model 2: further adjusted for income (<£18,000/£18,000–30,999/£31,000–51,999/£52,000–100,000/>£100,000), smoking and drinking habits (never/former/current), BMI (continuous), systolic BP (continuous), total cholesterol (continuous), high-density lipoprotein (continuous), serum creatinine (continuous), vitamin D supplement (yes/no), and the use of antihypertensive drugs (yes/no), lipid-regulating drugs (yes/no), and statins (yes/no).

## Table S9. Interaction effects of VDR polymorphisms on the inverse association between high 25(OH)D and cardiocerebrovascular outcomes in prediabetic individuals: restricted in Whites.

|  | **Genotype** | **Adjusted HR (95% CI)** | | **P _for_ _interaction_** |
| --- | --- | --- | --- | --- |
| **Myocardial infarction** | |  |  |  |
| rs731236 (*TaqI*) | GG / AA | 0.93 (0.74, 1.17) | 0.87 (0.75, 1.02) | 0.460 |
| rs7975232 (*ApaI*) | AA / CC | 0.87 (0.73, 1.03) | 0.91 (0.75, 1.12) | 0.858 |
| rs1544410 (*BsmI*) | TT / CC | 0.93 (0.74, 1.17) | 0.88 (0.75, 1.03) | 0.218 |
| rs2228570 (*FokI*) | GG / AA | 0.73 (0.63, 0.85) | 0.82 (0.65, 1.02) | **0.035** |
| **Stroke** |  |  |  |  |
| rs731236 (*TaqI*) | GG / AA | 0.56 (0.37, 0.83) | 0.95 (0.72, 1.24) | 0.059 |
| rs7975232 (*ApaI*) | AA / CC | 0.57 (0.43, 0.75) | 0.90 (0.66, 1.23) | 0.139 |
| rs1544410 (*BsmI*) | TT / CC | 0.57 (0.38, 0.84) | 0.94 (0.72, 1.24) | 0.060 |
| rs2228570 (*FokI*) | GG / AA | 0.67 (0.51, 0.88) | 0.65 (0.43, 0.99) | **0.016** |

HR=hazard ratio; CI=confidence interval; CVD=cardiovascular disease. Cox models were adjusted for sex (female/male), age (continuous), income (<£18,000/£18,000–30,999/£31,000–51,999/£52,000–100,000/>£100,000), smoking habits (never/former/current), drinking habits (never/former/current), BMI (continuous), systolic BP (continuous), total cholesterol (continuous), high-density lipoprotein (continuous), serum creatinine (continuous), and the use of vitamin D supplement (yes/no), antihypertensive drugs (yes/no), lipid-regulating drugs (yes/no), statins (yes/no), and hemoglobin A1c (continuous). HR indicated the risk for per one unit increment in serum 25(OH)D concentration.
